# Psychometric Properties of the Health Literacy Survey European Questionnaire-12

**DOI:** 10.1093/geroni/igab046.1987

**Published:** 2021-12-17

**Authors:** Suyeong Bae, Sung-Ji Park, Hee-Soon Woo, Ickpyo Hong

**Affiliations:** 1 Yonsei University, Wonju, Kangwon-do, Republic of Korea; 2 Wonkwang university, Wonkwang University/Iksan, Cholla-bukto, Republic of Korea

## Abstract

The importance of health literacy has increased as the public awareness of health has increased. Health Literacy Survey European Questionnaire-47 (HLS-EU-Q47) is a representative assessment tool for evaluating health literacy, and its psychometric properties have been examined in various countries. This study analyzed the item-level psychometric properties of a short version of the HLS-EU-Q47 using a Rasch measurement model. We collected 254 Korean adults who completed the 12 items of the HLS-EU-Q47 in hospital settings. We used confirmation factor analysis (CFA) to examine the unidimensionality assumption of the HLS-EU-Q12. We analyzed item fit, precision, and differential item functioning (DIF) across sex, age and education groups. The CFA model confirmed that HLS-EU-Q12 satisfies the unidimensionality assumption (CFI = 0.96, TLI = 0.96, RMSEA = 0.09) and no local independence in the 12 test items (residual correlations ranged from -0.16 to 0.19). The HLS-EU-Q12 demonstrated high reliability (Cronbach’s α = 0.90) and no DIF across sex, age and education groups (p > 0.05). The person strata by the instrument were 3.80, which is equivalent to a traditional reliability value of 0.87. In short, the study findings indicate that the HLS-EU-Q12 has good psychometric properties with the 254 Korean adults. Since the HLS-EU-Q12 can accurately and precisely evaluate the health literacy of Korean adults, this instrument could be used in clinical settings.

